# Dopamine Regulates Mobilization of Mesenchymal Stem Cells during Wound Angiogenesis

**DOI:** 10.1371/journal.pone.0031682

**Published:** 2012-02-15

**Authors:** Saurav Shome, Partha Sarathi Dasgupta, Sujit Basu

**Affiliations:** 1 Department of Signal Transduction and Biogenic Amines, Chittaranjan National Cancer Institute, Kolkata, West Bengal, India; 2 Department of Pathology, Ohio State University, Columbus, Ohio, United States of America; 3 Dorthy M. Davis Heart and Lung Research Institute, Ohio State University, Columbus, Ohio, United States of America; 4 Arthur G. James Comprehensive Cancer Center, Ohio State University, Columbus, Ohio, United States of America; Children's Hospital Boston & Harvard Medical School, United States of America

## Abstract

Angiogenesis is an important step in the complex biological and molecular events leading to successful healing of dermal wounds. Among the different cellular effectors of wound angiogenesis, the role of mesenchymal stem cells (MSCs) is of current interest due to their transdifferentiation and proangiogenic potentials. Skin is richly innervated by sympathetic nerves which secrete dopamine (DA) and we have recently shown that concentration of DA present in synaptic cleft can significantly inhibit wound tissue neovascularization. As recent reports indicate that MSCs by mobilizing into wound bed play an important role in promoting wound angiogenesis, we therefore investigated the effect of DA on the migration of MSCs in wound tissues. DA acted through its D_2_ receptors present in the MSCs to inhibit their mobilization to the wound beds by suppressing Akt phosphorylation and actin polymerization. In contrast, this inhibitory effect of DA was reversed after treatment with specific DA D_2_ receptor antagonist. Increased mobilization of MSCs was demonstrated in the wound site following blockade of DA D_2_ receptor mediated actions, and this in turn was associated with significantly more angiogenesis in wound tissues. This study is of translational value and indicates use of DA D_2_ receptor antagonists to stimulate mobilization of these stem cells for faster regeneration of damaged tissues.

## Introduction

Angiogenesis, the formation of new blood vessels from pre-existing ones is a normal physiological process and plays an important role in wound healing [1]–[2]. This complex and dynamic process further involves multiple cellular and molecular regulators, among which the roles of endothelial cells [1]–[2] and endothelial progenitor cells [3]–[5] have been well documented. However, recent attention has been drawn to the role of mesenchymal stem cells (MSCs) in wound angiogenesis and the healing process [6]–[10]. MSCs are multipotent stem cells present in adult bone marrow, umbilical vein and adipose tissue, and these adult stem cells have the capacity to proliferate and differentiate into different mesenchymal lineage cells [11]–[13]. Wound results in the release of various growth factors and cytokines and these molecules by acting as chemokines increase the mobility of MSCs from their sources, thereby facilitating migration of MSCs into the peripheral blood and from there into wound bed [14]–[16]. Accumulating MSCs at wounded sites accelerate the process of wound tissue angiogenesis, an essential physiological step for successful wound tissue repair by transdifferentiating into different cell types, which include endothelial cells, the principal structural component of wound tissue neovessels [8], [10], [13], [17]–[19]. In addition, these MSCs have the capacity to release various proangiogenic factors like vascular endothelial growth factor (VEGF) to support the growth, survival and differentiation of endothelial cells [9], [13], [17], [19]–[20].

Previous studies from our laboratory have conclusively demonstrated that endogenous catecholamine neurotransmitter DA by acting through its D_2_ receptors can significantly inhibit angiogenesis in malignant tumors [21]–[24]. Recent studies from our laboratory have also shown that DA by acting via its D_2_ receptors negatively influences the process of normal wound healing in a murine model of full thickness dermal wounds, and treatment with specific DA D_2_ receptor antagonist significantly accelerates the process of neovascularization in wound tissues leading to faster healing [25]. As recent reports indicate important roles of MSCs in wound angiogenesis, we therefore investigated whether DA can regulate this neovascularization process in normal wound tissue by influencing the mobilization of MSCs into wound site and their subsequent pro-angiogenic effects during wound healing.

## Results

### Treatment with specific DA D_2_ receptor antagonist following injury significantly increases number of MSCs (CD34^−^ CD45^−^ CD105^+^ cells) in peripheral blood

**Figure 1:**
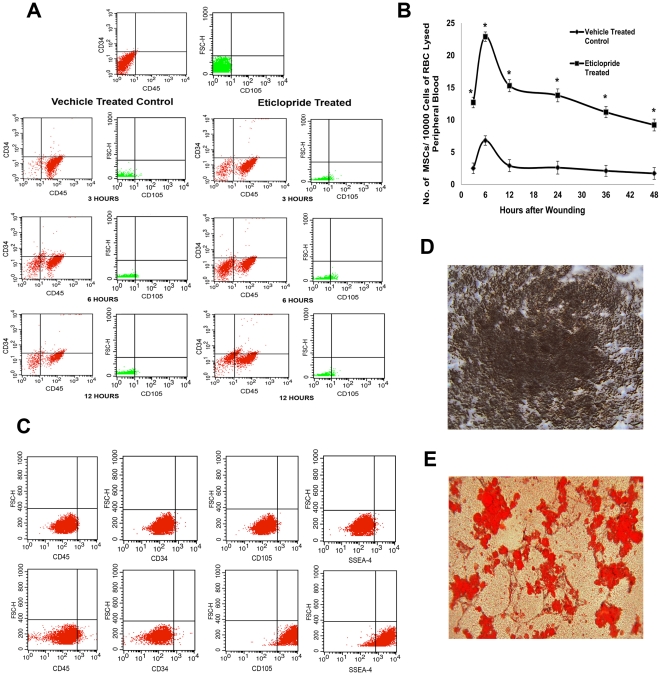
Treatment with dopamine (DA) D_2_ receptor specific antagonist significantly increased the number of circulating MSCs in peripheral blood of wound bearing mice. (**A**) Effect of eticlopride (DA D_2_ receptor specific antagonist) on the number of circulating MSCs in peripheral blood of wound bearing mice. Blood was collected from both the control and eticlopride treated wound bearing mice at different time intervals after injury, mixed with EDTA, treated with RBC lysis buffer and incubated with anti-CD45, anti-CD34 and anti-CD105 antibodies. Cells were initially gated to exclude dead cells and debris and then the CD34^−^ CD45^−^ cell population was selected and evaluated for presence of CD105, a specific surface marker of MSCs. Results are representative of six separate experiments each yielding similar results. (**B**) Graph showing that DA D_2_ receptor antagonist treatment have significant positive regulatory effect on MSC mobilization as significantly higher number of MSCs (CD34^−^ CD45^−^ CD105^+^ cells) was evident in peripheral blood of mice compared to vehicle treated controls at all time points after wounding. (*, P<0.05). (**C**) Characterization of MSCs by flow cytometry. Analysis with flow cytometer showed that over 95% cells of the total 3rd passage murine bone marrow cell populations were positive for both SSEA-4 and CD105 (specific surface markers of MSCs) and negative for CD34 and CD45, thus confirming their identity as MSCs. BM-MSCs were cultured in appropriate induction media to evaluate osteogenic and adipogenic differentiation potential of these stem cells and were confirmed by von Kossa staining (D) and Oil-red O staining (E) respectively. (**D**) To confirm osteogenic differentiation, mineralized deposits in the extracellular matrix were visualized by von Kossa staining. Differentiated cells were fixed with 4% paraformaldehyde, washed and then stained with 5% silver nitrate (Sigma) solution in the absence of light at room temperature for 30 minutes. Cells were then exposed to sunlight for 5 minutes and excess silver staining was removed by washing 2–3 times with a 5% sodium thiosulfate solution and ultimately washed with distilled water. (**E**) Adipogenic differentiation of cultured MSCs was confirmed by Oil-red O staining. Cells were fixed with 4% paraformaldehyde and washed with 60% isopropanol. After completely drying the flasks Oil Red O working solution were added and stained for 15 minutes. Then all Oil Red O were removed and distilled water was added immediately to wash 2 times. Finally, the flasks were viewed under microscope and photographed. Original magnifications, ×200.

Recent studies from our laboratory have shown that treatment with specific DA D_2_ receptor antagonist significantly accelerates the time of wound healing in a murine model of full thickness normal dermal wounds, and this healing in turn is associated with increased angiogenesis in wound tissues [25]. Mobilization of MSCs into wound bed and their subsequent active participation in wound tissue neovascularization are critical steps towards successful wound healing [6]–[8], [10], [13], [17]–[19]. Therefore, in the present investigation to explore the regulatory role of DA D_2_ receptors, if any, on mobilization of MSCs into wound site, the status of circulating MSCs in peripheral blood of both control and eticlopride treated back skin-injured mice had been compared at different time intervals by flow cytometry to determine the effect of inhibitory action of DA D_2_ receptors on the profile of circulating MSCs. The results showed that treatment with DA D_2_ receptor antagonist eticlopride significantly increased the numbers of circulating MSCs (immunophenotypically CD34^−^ CD45^−^ CD105^+^ cells) [26] in peripheral blood of wound bearing mice in comparison to vehicle treated controls at different time intervals (3, 6, 12, 24, 36 and 48 hours after wounding) (Fig. 1A and 1B). In both control and treated animals, the number of circulating MSCs showed a sharp increase that reached peak at 6 hour after creation of wounds (Fig. 1A). However, this increase in the numbers of circulating MSCs were significantly higher in DA D_2_ receptor antagonist treated mice in comparison to vehicle treated controls at all time points (Fig. 1B). Similar results were also observed following treatment with another DA D_2_ receptor specific antagonist domperidone (results not shown). It is to be noted here that treatment with other DA receptor antagonists (D_1_, D_3_, D_4_ and D_5_) had no significant effects on the mobilization of MSCs (results not shown). This data confirmed that the action of DA was specific and was mediated through its D_2_ receptors present in MSCs.

### CD34^−^ CD45^−^ CD105^+^ Cells showed osteogenic and adipogenic differentiation potential *in vitro*

Mesenchymal stem cells are multipotent in nature and have the unique ability to differentiate into various cell types among which osteogenic and adipogenic differentiation are considered as hallmarks of these adult stem cells [26]. Therefore, to confirm the identity of CD34^−^ CD45^−^ CD105^+^ cells as MSCs, their osteogenic and adipogenic differentiation potential were evaluated. Using lineage cell depletion kit (Miltenyi Biotec, Germany), mesenchymal lineage cells were isolated from total bone marrow cells by depletion of cells of haematopoietic lineage, cultured in MSC media and expanded *in vitro* for three passages [27]. Flow cytometry demonstrated that over 95% of the total cells were phenotypically CD34^−^ CD45^−^ CD105^+^ in nature, characteristics of MSCs (Fig. 1C). These CD34^−^ CD45^−^ CD105^+^ cells also expressed SSEA-4, a stage-specific embryonic antigen that identifies adult mesenchymal stem cell population from the bone marrow [28]. These cells were then cultured in appropriate induction media to induce osteocytes and adipocytes [29]. In the initial osteogenic culture, no mineralized cell was detected. However, after five weeks, mineralized cells positive for von Kossa staining showing calcium deposition were observed (Fig. 1D) thus indicating osteogenic differentiation of these cells. Similarly one week after adipogenic induction, formation of intracellular lipid vacuoles were detected in these cells. Thereafter, lipid accumulation increased along with the inductive periods, which was chemically stained by Oil Red O, a specific staining, which identifies lipid droplets in adipocytes, thereby, confirming adipocytic nature of these cells (Fig. 1E).

### Murine MSCs express DA D_2_ receptors

**Figure 2:**
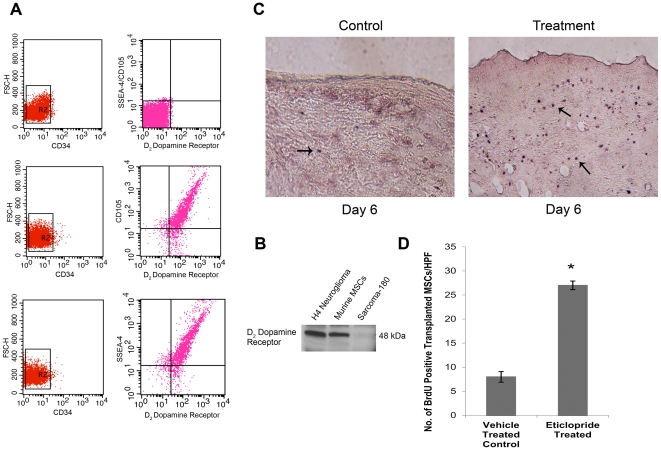
Treatment with dopamine (DA) D_2_ receptor antagonist significantly accelerated mobilization of exogenously transplanted MSCs towards wound bed. (**A**) Flow cytometric analysis of DA D_2_ receptors in murine MSCs. To confirm that CD34^−^ CD45^−^ CD105^+^ SSEA-4^+^ cells express DA D_2_ receptors, *in vitro* expanded Lin^neg^ bone marrow cell population (containing CD45^−^, CD11b^−^ cell populations) were initially gated to exclude dead cells and debris and then the CD34^−^ cell population were selected and these CD34^−^ CD45^−^ cells were evaluated for presence of CD105, SSEA-4 and DA D_2_ receptors. Results showed that almost 86% cells of the total MSC population (both CD34^−^ CD45^−^ CD105^+^ cells and CD34^−^ CD45^−^ SSEA-4^+^ cells) express DA D_2_ receptors on their surfaces. (**B**) Western blot analysis of DA D_2_ receptors in murine BM-MSCs. H4 neuroglioma cell line and sarcoma-180 (S-180) tumor cells were used as positive and negative controls, respectively. (**C**) Effect of DA D_2_ receptor antagonist treatment on mobilization of exogenously transplanted MSCs towards wound site. MSCs were labeled with BrdU in culture and injected into the tail vein of both control and eticlopride treated back skin-injured mice. After completion of eticlopride treatment, at 6th day the skin from the wound area was collected and analyzed by immunohistochemistry using BrdU labeling and detection kit (Roche Applied Science). Significantly higher number of BrdU positive transplanted MSCs are located in the wound bed of eticlopride treated group than vehicle treated control group, showing DA D_2_ receptor antagonist treatment has significant positive effect on mobilization of exogenously transplanted MSCs towards wound site. Original magnifications, ×200. Results are representative of six separate experiments each yielding similar results. (**D**) Graphical representation showing significantly higher number of exogenously transplanted MSCs (BrdU positive cells) in wound bed of eticlopride treated groups compared to vehicle treated controls at day 6 post wounding (*, P<0.05). Number of transplanted MSCs cells was measured by counting the number of BrdU positive cells in 10 randomly chosen high power microscopic fields within the sections.

To confirm the role of DA D_2_ receptors in the migration of MSCs into wound tissue, it was necessary to know whether these cells expressed DA D_2_ receptors. Therefore, the presence of DA D_2_ receptor was investigated by western blot and flow cytometry. Western blot analysis of *in vitro* expanded murine MSCs showed presence of DA D_2_ receptors in these cells, which was further confirmed by flow cytometry analysis (Fig. 2A and 2B). Flow cytometry of CD45^−^ CD34^−^ CD105^+^ SSEA-4^+^ cells confirmed that almost 86% cells of the total MSC population expressed DA D_2_ receptors on their surfaces (Fig. 2A).

### Treatment with DA D_2_ receptor antagonist significantly accelerated mobilization of exogenously transplanted MSCs towards wound bed

As it was evident that treatment with DA D_2_ receptor antagonist increased the number of circulating MSCs in wound bearing mice (Fig. 1A and 1B), therefore experiment was undertaken to determine mobilization of these circulating MSCs cells into the wound bed, the site where these cells are required to migrate in order to mediate wound angiogenesis and tissue repair. To investigate the effect of DA D_2_ receptor antagonist on the homing of MSCs into wound site, bone marrow derived and expanded MSCs (CD34^−^ CD45^−^ CD105^+^ cells) were labeled with BrdU in culture and were then injected i.v. via the tail veins into both control and eticlopride treated back skin-injured mice on day 1 after wounding. These cells were injected 2 hrs after DA D_2_ receptor antagonist treatment. The transplanted MSCs in the wound bed were identified by BrdU immunoreactivity. Our study revealed significantly higher number of BrdU labeled MSC transplants in DA D_2_ receptor antagonist treated wound tissues than vehicle treated controls on day 6 after creation of wound and completion of 4 day treatment schedule (10 mg/kg/day) of eticlopride (Fig. 2C and 2D).

### Increased integration of fluorophore-labeled (CM-Dil) transplanted MSCs into blood vessels in wound bed following DA D_2_ receptor antagonist treatment

**Figure 3:**
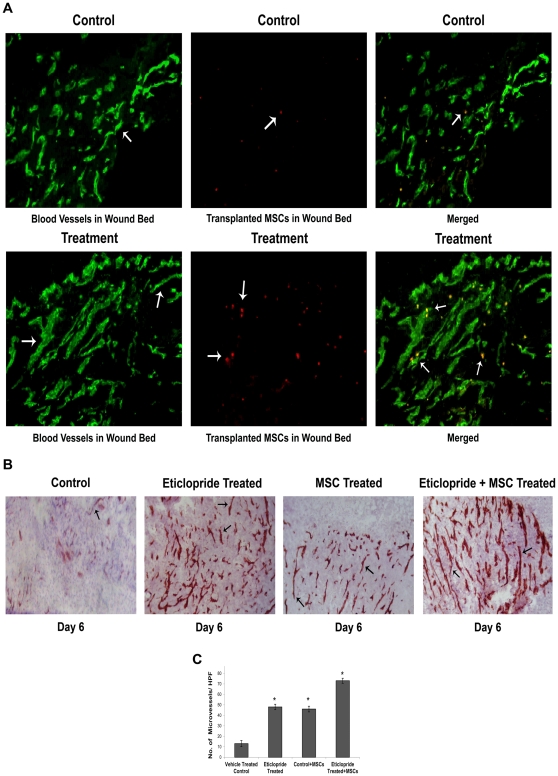
Dopamine (DA) D_2_ receptor antagonist treatment stimulate incorporation of MSCs into neovessels of wound bed and subsequent angiogenesis in wound bed. (**A**) Effect of eticlopride treatment on incorporation of transplanted MSCs into newly formed blood vessels in wound bed. MSCs were labeled with CM-Dil and injected into the tail vein of both control and eticlopride treated back skin-injured mice. At day 6, the skin from the wound area was collected and sectioned with cryomicrotome. Frozen sections were immunostained with anti-CD31 antibody and FITC-conjugated secondary antibody and analyzed to determine the extent of incorporation of transplanted MSCs into blood vessels. Here co-localization study showed that exogenously transplanted MSCs, labeled with fluorescent dye CM-Dil (red in colour), had integrated into the newly formed blood vessels (green in colour) in wound site in much greater number following DA D_2_ receptor antagonist treatment than vehicle treated controls. Original magnifications, ×200. (**B**) Effect of MSC transplantation on formation of new blood vessels in wound bed. Immunohistochemical staining of CD31, a specific endothelial cell surface marker, shows significantly greater number of microvessels in wound tissue sections of DA D_2_ receptor antagonist treated mice in comparison to vehicle treated controls at day 6 post wounding. Treatment with MSCs significantly increases microvessel density in wound bed in comparison to saline treated controls. However, eticlopride treatment along with MSCs transplantation is most effective in increasing angiogenesis in wound tissue. Original magnifications ×200. (**C**) Graphical representation showing microvessels density in wound tissue sections of different experimental groups at day 6 after post wounding (*, P<0.05). Microvessel density (CD31 positive cells) in wound bed was measured by counting the number of microvessels in 10 randomly chosen high power microscopic fields within the sections.

Our previous results had shown that treatment with DA D_2_ receptor antagonist eticlopride significantly increased the wound healing process by stimulating neovascularization [25]. Interestingly, our present results also revealed that this treatment was associated with increased number of migrated MSCs into wound sites (Fig. 2C and 2D). Therefore, we investigated whether this increased number of mobilized MSCs in wound tissue following DA D_2_ receptor antagonist treatment was also associated with increased incorporation of these cells into the neovessels of healing wounds. Co-localization studies showed that on day 6 after wounding and completion of 4 day of eticlopride treatment schedule, exogenously transplanted MSCs, labeled with fluorescent dye CM-Dil (red in colour), had incorporated into the newly formed blood vessels in wound site (CD31 positive cells, green in colour) in considerably higher numbers than vehicle treated controls (Fig. 3A).

Furthermore our results also demonstrated that treatment with eticlopride in combination with transplanted MSCs resulted in significantly more angiogenesis in wound tissues in comparisons to the vehicle treated controls or animals treated with eticlopride or MSCs alone (Fig. 3B and 3C).

### DA through its D_2_ receptors regulate VEGF induced migration of MSCs *in vitro*

*In vivo* results from the present investigation had shown that exogenously transplanted BrdU labeled MSCs migrated to the wound bed in significantly higher numbers in DA D_2_ receptor antagonist eticlopride treated wound bearing mice than vehicle treated controls (Fig. 2C). It was also evident from co-localization study that greater numbers of these cells had integrated into the neovessels of the wound bed (Fig. 3A).

Several recent reports have indicated that VEGF is the predominant growth factor, which regulates wound angiogenesis [30]–[33] and chemotactic migration of MSCs both *in vitro* and *in vivo* [14], [15], [31], [34]–[37], therefore further experiments were designed *in vitro* to determine the direct effect of VEGF on the migration of murine MSCs.

**Figure 4:**
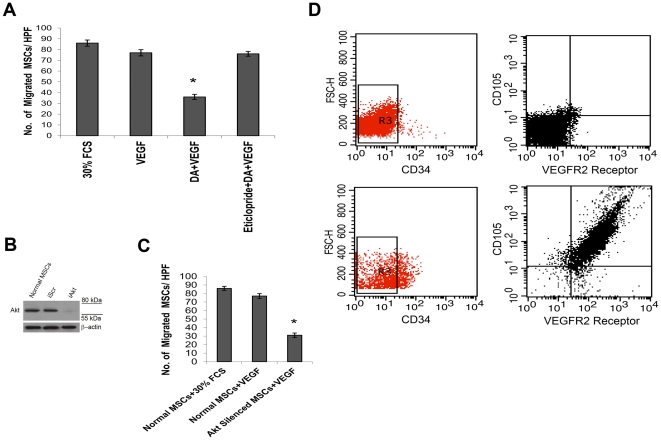
VEGF regulates migration of murine BM-MSCs through activation of Akt. (**A**) Effects of VEGF on *in vitro* migration of MSCs in transwell chambers. After incubation with MSCs at 37°C for 24 hours, VEGF was added. After overnight incubation cells remaining on the upper face of the filters were removed with a cotton wool swab and migrated cells that remained on the lower face of the filters were stained with Gill's Haematoxylin, counted and photographed. Numbers of migrated cells were counted in 10 high-power fields (HPFs) after subtraction of the basal migration observed in the presence of DMEM alone (without presence of any growth factor). Dopamine (DA) significantly inhibits VEGF induced migration of MSCs (*, P<0.05), whereas when cells were pre-treated with 100 µM eticlopride (a specific DA D_2_ receptor antagonist), the inhibitory effect of 1 µM DA on VEGF mediated mobilization was abrogated. Results are representative of six separate experiments each yielding similar results. (**B**) Silencing of Akt in MSCs by siRNA transfection. After 48 hours of transfection, no expression of Akt was found in MSCs transfected with Akt siRNA (iAkt) whereas control MSCs in which control siRNA containing scrambled sequence (iScr) was transfected showed similar expression of Akt as normal non-transfected MSCs. (**C**) Effect of Akt silencing on VEGF induced migration of MSCs in a chemotaxis assay. Numbers of migrated cells were counted in 10 high-power fields (HPFs) after subtraction of the basal migration observed in the presence of DMEM alone (without presence of any growth factor). Silencing of Akt significantly inhibits VEGF induced migration of MSCs (*, P<0.05). Results are representative of six separate experiments each yielding similar results. (**D**) Flow cytometric analysis of VEGFR-2 receptors in murine MSCs. To confirm that CD34^−^ CD45^−^ CD105^+^ cells express VEGFR-2 receptors, CD34^−^ cells were selected from *in vitro* expanded Lin^neg^ bone marrow cell population (containing CD45^−^, CD11b^−^ cell populations) and these CD34^−^ CD45^−^ cells were evaluated for presence of CD105 and VEGFR-2 receptors. Results showed that almost 89% cells of the total MSC population (CD34^−^ CD45^−^ CD105^+^ cells) express VEGFR-2 receptors on their surfaces.

The results showed that the numbers of migrated MSCs (CD34^−^ CD45^−^ CD105^+^ cells) in the presence of medium alone (DMEM without FCS) was very low and it increased in the presence of 30% FCS or VEGF (10 ng/ml) (Fig. 4A). This result corroborates well with the critical role of VEGF in regulating chemotaxis of MSCs into wound bed as reported by several other studies [15], [31], [34]–[37]. Therefore, further experiments were designed *in vitro* to evaluate the role of DA on VEGF induced migration of murine MSCs.

Dermal tissues are richly innervated by sympathetic nerve fibers in which DA is a major catecholamine neurotransmitter found in the extracellular fluid surrounding neuronal synapses [38]–[39]. Therefore in the present investigation, the effect of physiological concentration of DA (1 µM as observed in neuronal synapses) [40] was evaluated in regulating VEGF induced migration of MSCs *in vitro*.

Addition of 1 µM DA in the medium significantly inhibited VEGF induced migration of MSCs, whereas, when these cells were pre-treated with 100 µM eticlopride (a specific DA D_2_ receptor antagonist), the inhibitory effect of DA on this growth factor mediated mobilization was abrogated (Fig. 4A). Thus, the *in vitro* results showed that physiological concentration of DA as observed in neuronal synapse [40] inhibits VEGF induced migration of MSCs. Furthermore, this *in vitro* result also correlated well with our previous *in vivo* observations of eticlopride, a DA D_2_ receptor antagonist-mediated increased mobilization of MSCs into wound bed.

### DA regulates VEGF induced migration of murine MSCs by inhibiting Akt phosphorylation

Because neurotransmitter DA by acting through its D**_2_** receptors significantly inhibited VEGF induced migration of murine MSCs *in vitro* (Fig. 4A), we next elucidated the regulatory role of DA in influencing the key signaling pathway for VEGF induced mobilization of MSCs into the wound site. Since recent experimental results have shown that activation of Akt increases the migratory activity of MSCs [41]–[44], experiments were therefore designed to dissect the regulatory role of Akt in VEGF induced migration of MSCs in mice. siRNA transfection was utilized to knock-down (silence) Akt in MSCs. After 48 hours, western blot analysis was performed to confirm silencing in Akt siRNA transfected cells (iAkt) (Fig. 4B).

The role of Akt kinase on growth factor induced mobilization of murine MSCs was then evaluated by *in vitro* migration assay of siRNA transfected (Akt-silenced) MSCs against chemotactic activity of 10 ng/ml VEGF. Silencing of Akt significantly inhibited VEGF induced migration of MSCs (Fig. 4C). Furthermore, flow cytometry analysis showed that murine BM-MSCs also express VEGFR-2 receptors on their surfaces (Fig. 4D), which corroborated well with previous observations [45]–[46] thus showing that VEGF considerably influences mobilization of murine MSCs by activating Akt kinase through its VEGFR-2 receptors present on these cells.

**Figure 5:**
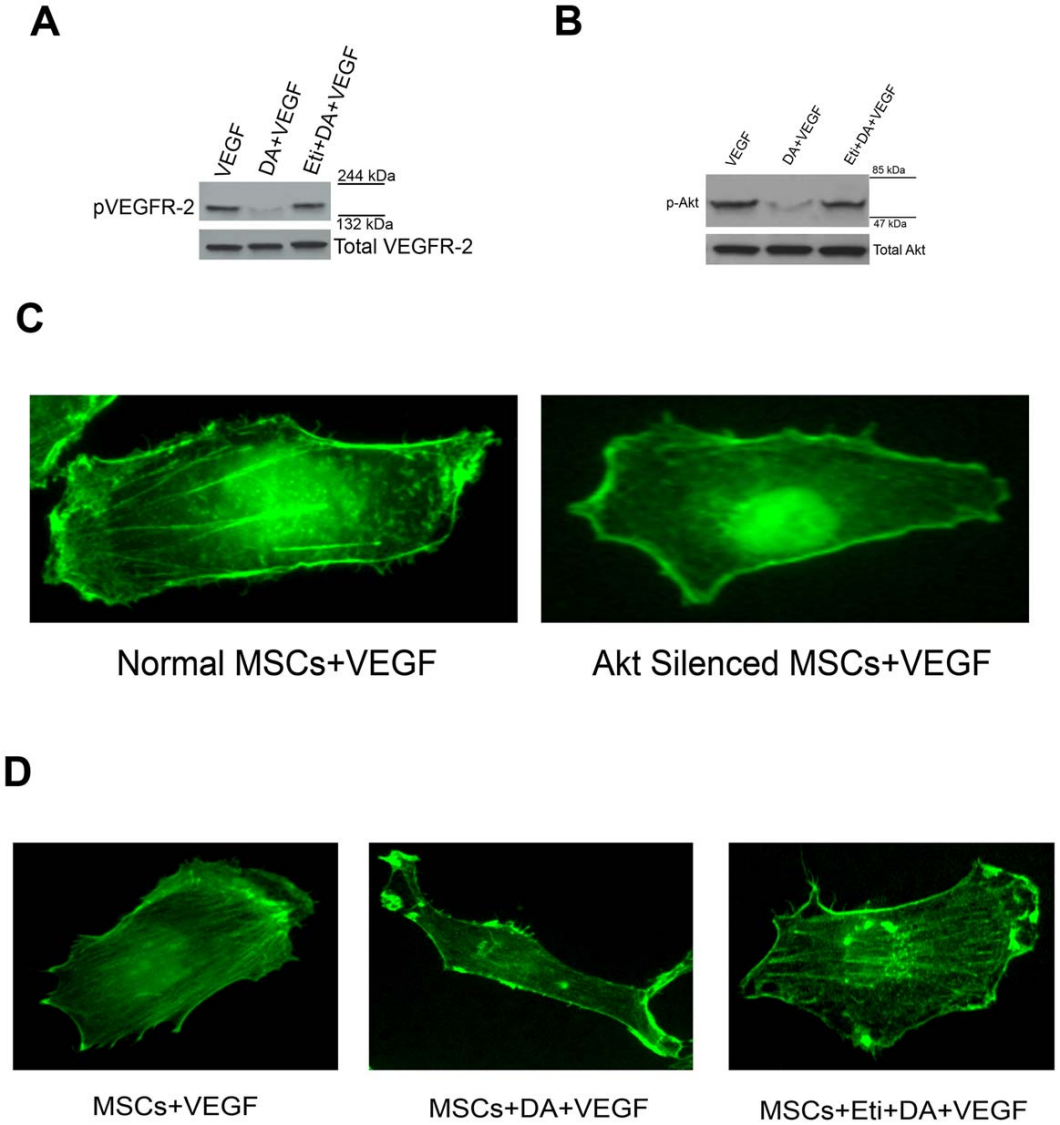
Dopamine (DA) through its D_2_ receptors inhibits VEGF induced migration of BM-MSCs by regulating phosphorylation of VEGFR-2 and Akt and actin polymerization. (**A and B**) Effect of DA on VEGF induced phosphorylation of VEGFR-2 and Akt in murine MSCs. Lane 1: Cells stimulated with VEGF (10 ng/ml). Lane 2: Cells pretreated with 1 µM DA before being exposed to VEGF (10 ng/ml). Lane 3: Cells treated with 100 µM eticlopride followed by DA and VEGF. Addition of DA significantly inhibited VEGF induced phosphorylation of both VEGFR-2 receptors and its downstream target Akt when compared with VEGF treated control. Pre-treatment with eticlopride, abrogated DA induced dephosphorylation of both VEGFR-2 and Akt. However, expression of total VEGFR-2 and total Akt remained unchanged. Results are representative of six separate experiments each yielding similar results. (**C**) In actin polymerization assay, VEGF induced MSCs showed significant polymerization of actin cytoskeleton leading to formation of F-actin. However, no such alterations were observed in case of Akt silenced MSCs which showed strikingly lower migratory activity against VEGF than normal MSCs. (**D**) Effect of DA on VEGF induced actin polymerization in MSCs. Treatment with 1 µM DA had significant inhibitory effect on actin polymerization dynamics in MSCs when compared to VEGF treated control. However, pre-treatment with specific DA D_2_ receptor antagonist eticlopride (100 µM) abrogated the DA induced changes in the actin polymerization dynamics of the VEGF induced MSCs. Original magnifications, ×1000.

We next elucidated whether DA exerted its inhibitory effects on the mobilization of MSCs by suppresing the activation of Akt in these cells. The activation of DA receptors in MSCs by 1 µM of DA significantly inhibited VEGF (10 ng/ml) induced phosphorylation of both VEGFR-2 and its downstream signaling molecule Akt (Fig. 5A and 5B). However, these actions of DA were abrogated following treatment with 100 µM eticlopride, a DA D_2_ receptor antagonist (Fig. 5A and 5B). In contrast, no changes were observed in the expression of total VEGFR-2 and total Akt. These results indicated that DA through its D_2_ receptors significantly inhibited VEGF induced migration of murine MSCs by dephosphorylating both VEGFR-2 receptors and its downstream target Akt kinase in these stem cells.

### DA regulates polymerization of actin filaments in migrating MSCs

Reorganization and active regulation of actin cytoskeleton is an important event in the migratory response of cells to growth factors, and phosphorylation of Akt had been implicated to induce this actin polymerization [43]–[44].

In our actin polymerization assay, VEGF stimulated MSCs which showed high migratory activities [Fig. 4A], was also associated with changes in the polymerization dynamics of this protein leading to formation of F-actin [Fig. 5C]. However, no such alterations were observed in Akt silenced MSCs [Fig. 5C], which also showed strikingly lower migratory activity against VEGF in comparison to normal MSCs [Fig. 4C]. These results indicated that Akt regulated the mobilization of MSCs by changing polymerization dynamics of actin cytoskeleton.

Furthermore, treatment with 1 µM of DA, which significantly inhibited VEGF induced migration of MSCs by suppressing phophorylation of both VEGFR-2 and its downstream signaling molecule Akt, was also associated with significant actin depolymerization effect leading to the inhibition MSCs migration (Fig. 5D). In contrast, pre-treatment with specific DA D_2_ receptor antagonist eticlopride abolished this DA mediated inhibitory changes in the actin polymerization dynamics of VEGF treated MSCs. These results indicated that DA regulated inhibition of VEGF induced actin polymerization and migration of MSCs were specifically mediated through its D_2_ receptors.

## Discussion

Angiogenesis is necessary for successful wound healing and defects in this process leads to delayed healing as observed in different types of chronic non-healing wounds. During cutaneous wound repair, the neovessels provide nutrition and oxygen to the growing tissues and aids in the formation of the provisional wound matrix. This dynamic physiological process is temporally and spatially regulated by interactions between different cell types and other factors present in the wound microenvironment [1]–[2]. Among the various cellular effectors, the roles of endothelial cells and endothelial progenitor cells are well established in the process of angiogenesis [1]–[5]. In pathological conditions like malignant tumors, DA can significantly inhibit tumor angiogenesis by inhibiting VEGF induced proliferation and migration of both adult and progenitor endothelial cells [21]–[24]. We have recently demonstrated that DA can inhibit angiogenesis in normal wound tissue by inhibiting the expression of HoxD3 and its target α5β1 integrin in adult endothelial cells [25]. DA acted through its D_2_ receptors [21]–[24]. However, current attention has been drawn to the role of MSCs in the formation of neovessels in wound tissues.

Mesenchymal stem cells are either absent or present in significantly low numbers in the steady state conditions in circulation. However, their numbers considerably increase after trauma or injury and this is associated with increased level of plasma VEGF [14]–[16]. These circulating cells then migrate to the site of the injury by the process of chemotaxis and subsequently incorporate into the newly formed vessels to be transdifferentiated into endothelial cells [8], [10]. Several studies have indicated that MSCs can differentiate into endothelial cells and are capable of inducing neoangiogenesis [8], [10], [47]–[48]. In addition, these stem cells also release several pro-angiogenic molecules like VEGF to recruit inflammatory and progenitor cells to make the wound tissue microenvironment conducive for neovessel formation and subsequent healing [9], [13], [17], [19]–[20], [49]. These reports thus clearly indicate the importance of MSCs in wound tissue angiogenesis.

The bone marrow and the adipose tissue are considered to be the principal sources of MSCs [11]–[13], [17]–[20] and both these tissues are richly innervated by the sympathetic nerves [50]–[53]. Furthermore, substantial amount of dopamine is present in the bone marrow niche as well as adipocytes [24], [54]. We have previously reported that DA by acting through its DA D_2_ receptors can inhibit the activity of MMP-9, a potent regulator cell mobilization from bone marrow to peripheral circulation [24]. Therefore, in the present study, the increased numbers of MSCs in the circulation of specific DA D_2_ receptor antagonist treated wound bearing mice may be due to inhibition of DA induced suppression of adipose tissue and/or bone marrow MMP-9 activity. Our study also for the first time demonstrates the presence of DA D_2_ receptors in the bone marrow-derived MSCs.

In the present investigation, *in vivo* results showed increased incorporation of exogenously transplanted MSCs into wound bed after eticlopride treatment. Furthermore, it was well associated with significantly enhanced angiogenesis. These results corroborate well with other reports that indicate significant pro-angiogenic effect of these stem cells [6]–[10], [48]–[49]. This present study also indicates that the physiological concentration of DA (1 µM) [40] present in synaptic clefts can inhibit the incorporation of exogenously transplanted MSCs into wound bed. Several recent reports have indicated that VEGF is the prime growth factor that regulates wound angiogenesis and migration of MSCs both in vitro and in vivo [14], [30]–[47], therefore in the present investigation while exploring the effect of DA on MSC mobilization, VEGF was used *in vitro* to induce chemotaxis.

Furthermore, phosphorylation of Akt induced by different growth factors has been shown to be one of the major signaling pathways regulating migration of MSCs [41]–[44]. In the present investigation, DA-mediated inhibition of MSCs migration was observed due to inhibition of Akt phosphorylation in murine MSCs. However, this effect of DA was abrogated after treatment with specific DA D_2_ receptor antagonist, thereby indicating that the action of DA was mediated through its DA D_2_ receptors. Since Akt regulates mobilization of MSCs by influencing polymerization dynamics of actin cytoskeleton, leading to actin polymerization and subsequently MSCs migration [43]–[44], we also determined the effect of DA on actin polymerization dynamics of MSCs. Our results indicated that DA mediated inhibition of MSC mobilization was associated with the inhibition of actin polymerization in these cells, and treatment with specific DA D_2_ receptor antagonist abrogated these effects. This further confirmed that the inhibitory effect of DA on MSC migration was specifically mediated through its D_2_ receptors. In summary, activation of DA D_2_ receptors in MSCs resulted in the inhibition of VEGF induced phosphorylation of Akt, which in turn suppressed actin polymerization and subsequent migration of these stem cells. Recent studies by other groups have also shown that activation of the Akt increases the migratory activity of human MSCs, whereas treatment with PI3K inhibitor blocks activation of Akt and thereby, significantly inhibits migration of these cells [44]. Our results also demonstrated that like human MSCs, Akt also played a critical role in regulating migration of murine MSCs.

This information generated from our present study indicate for the first time that physiological concentration of DA can negatively regulate mobilization of MSCs into wound sites and consequently influence angiogenesis in wound tissues. This novel finding is of clinical significance as DA D_2_ receptor specific antagonists being presently used for the treatment of other disorders may be utilized for accelerating mobilization of MSCs to expedite angiogenesis and hence tissue regeneration.

## Materials and Methods

### Experimental wound model and treatment

All animal experiments were performed after approval by the Institutional Animal Care and Use Committees. The experiments were carried out in full thickness dermal wound bearing normal Swiss mice (4–6 weeks and weighing 22–25 g). The animals were anesthetized with intraperitoneal (i.p.) injection of 100 µl solution containing ketamine and xylazine mixture (2.215 and 0.175 mg, respectively; Sigma). The dorsal hair of the mouse was shaved and disinfected with an alcohol (70% ethanol) swab and two excisional wounds were created at the same cranial-caudal level on the dorso-medial back of each animal using an 8 mm dermal punch biopsy. At the end of the surgical procedure, cages were placed on a heating pad until mice fully recovered from anesthesia [25].

Following creation of wounds, the wound bearing mice were divided into two groups. Immediately after wounding, mice of the treatment group were injected i.p. with 10 mg/kg of eticlopride, specific DA D_2_ receptor antagonist (Sigma) in 300ìl normal saline and continued for four consecutive days at an interval of 24 hours. The control group received similar volume of normal saline only [25]. The experiments were also repeated with another specific DA D_2_ receptor antagonist domperidone (Sigma).

### Isolation, purification and expansion of MSCs from mouse bone marrow

MSCs were separated from bone marrow of normal Swiss mice by using MACS cell sorting system of Miltenyi Biotec (Germany). Bone marrow cells were collected by flushing the femurs and tibias from healthy female mice (6 weeks old). Cells obtained from bone marrow were incubated with a cocktail of biotin-conjugated monoclonal antibodies to remove the hematopoetic cells and then separated by anti-biotin microbeads-conjugated secondary antibody. The Lin^neg^ fraction (containing pure Mesenchymal lineage cells) was cultured in murine mesenchymal stem cell expansion medium (Millipore, USA). After 48 hours, the non-adherent cells were removed and fresh medium was added to the cells. Medium was changed every 2 or 3 days. The adherent spindle-shaped cells were further propagated for three passages [27].

### Osteogenic and adipogenic differentiation of *in vitro* expanded MSCs

Osteogenic differentiation was induced by culturing confluent mouse MSCs in α-minimum essential medium (α-MEM) supplemented with 10% FCS, 10 mM β-glycerophosphate, 50 µg/ml ascorbic acid 2-phosphate and 10 nM dexamethasone for 14–28 days. The media was changed every 3–4 days. Osteogenic differentiation of murine MSCs was confirmed by staining mineralized deposits in the extracellular matrix with von Kossa staining which stains calcium deposited in ECM [29].

Adipogenic differentiation was induced by culturing confluent mouse MSCs in α-minimum essential medium (α-MEM) supplemented with 10% FCS, 0.5 mM IBMX (3-isobutyl-1-methylxanthine), 10 µg/ml insulin and 1 µM dexamethasone (all from Sigma) for 21 days. The media was changed in every 3–4 days. Adipogenic differentiation of murine MSCs was confirmed by Oil-red O staining which stains lipid droplets within adipocytes [29].

### Flow cytometry

Antibodies used for flow cytometry analysis were anti-CD45 (FITC-conjugated conjugated; eBioscience), anti-CD34 (APC-conjugated; eBioscience), anti-CD105 (Phycoerythrin-conjugated; eBioscience), anti-SSEA-4 (Phycoerythrin-conjugated; R & D Systems), mouse anti-D_2_ DA receptor antibody (Santa Cruz), rat anti-mouse IgG-FITC (eBioscience) and anti-VEGFR-2 antibody (FITC-conjugated; BD biosciences). In order to determine the numbers of circulating MSCs in peripheral blood after injury, blood was collected from both control and DA D_2_ receptor antagonist treated wound bearing mice at different time intervals (3, 6, 12, 24, 36 and 48 hours after injury) by nicking the tip of the tail. Each time 400 µl blood was taken and mixed with anti-coagulant to prevent blood clotting. Then the collected blood samples were treated with RBC lysis buffer to remove RBCs and then incubated with different antibodies to evaluate the number of MSCs in circulation. Cells were initially gated to exclude dead cells and debris, and then the CD34^−^ CD45^−^ cell population was selected and evaluated for the presence of CD105, a surface marker of MSCs. According to the International Society for Cellular Therapy (ISCT), MSCs are positive for CD105 and negative for CD34 and CD45 [26]. Thus, the *in vitro* expanded spindle shaped Lin^neg^ cells (containing pure mesenchymal lineage cells) were examined for the presence or absence of specific surface markers to confirm their identity. In addition, the presence of DA D_2_ receptors and VEGFR-2 receptors on their surfaces were also determined in these cells by flow cytometry. Analyses were considered informative when adequate numbers of events (after acquisition of 10,000 cells per sample) were collected in the gated cells. Percentage of positive cells was finally determined after comparing them with matched isotype controls [25].

### Detection of DA D_2_ receptors in MSCs by western blot

*In vitro* expanded MSCs were lysed and the protein extracts from these cells were subjected to SDS-PAGE and then blotted onto PVDF membranes (Millipore). Mouse anti-D_2_ DA receptor IgG and goat anti-mouse IgG HRP conjugated (Santa Cruz) were used as primary and secondary antibodies respectively. Antibody reactive bands were detected by enzyme - linked chemiluminescence (Pierce) [25].

### Examination of the role of DA D_2_ receptor antagonist on homing of exogenously transplanted MSCs to the wound bed

Passage three MSC cultures were maintained at 50% confluence. 5-Bromo-2′-deoxy-uridine (BrdU) labeling reagent (Roche Applied Science) was added into the culture medium (1∶1000) 72 hours before transplantation. The BrdU labeled MSCs were then harvested by trypsinization and resuspended in normal saline for injection. On the first day after wounding, 10^6^ live MSCs were injected into the tail vein of both control and DA D_2_ receptor antagonist treated back skin-injured mice after 2 hours of DA D_2_ receptor antagonist injection. Thereafter, on completion of treatment schedule on day 6, the skin from the wound area was collected and the number of BrdU labeled MSCs transplants were determined immunohistochemically by using BrdU labeling and detection kit (Roche Applied Science) [55].

### Determination of the incorporation of exogenously transplanted MSCs into newly formed blood vessels in wound bed

Lipophilic tracer (CM-Dil; Invitrogen Bioservices) was used to label MSCs [56]–[57]. The MSCs were trypsinized from their passage 3 cultures and incubated in the working solution of CM-Dil (15 µM CM-Dil solution in HBSS) for 5 minutes at 37°C and then for an additional 15 minutes at 4°C. After labeling, cells were washed twice with PBS and resuspended in ice-cold normal saline for injection. On day 1 after wounding, 10^6^ live labeled MSCs were injected into both control and DA D_2_ receptor antagonist treated back skin-injured mice via tail vein, two hours after injection of DA D_2_ receptor antagonist. On day 6, the wound tissues were collected, washed in PBS and fixed in OCT compound at −20°C for 1 hour, and cryostat sections were cut from the mid-portion of the wounds with a cryomicrotome. Frozen sections were immunostained with anti-CD31 antibody (Rat anti-mouse; BD Pharmingen, USA) and FITC-conjugated secondary antibody (Goat anti-rat; Millipore, USA) or biotin conjugated secondary antibody (Rabbit anti-rat IgG; Millipore, MA) and then analyzed either to determine incorporation of CM-Dil labeled transplanted MSCs into the newly formed blood vessels in wound bed or microvessel density using ABC staining kit (Vector Laboratories) and Nova-Red substrate solution (Vector Laboratories) [25].

### *In vitro* Migration assay of MSCs

Migration assays were performed in transwell dishes (Corning Costar) 6.5 mm in diameter with 8 µm pore filters. The upper side of the transwell filter was coated for 1 hour at 37°C with 0.1% (wt/vol) bovine gelatin (Sigma-Aldrich) in PBS. After incubation with MSCs (5×10^5^ cells) at 37°C for 24 hrs, 10 ng/ml VEGF (ProSpec) was added to the bottom chamber. Migration observed in the presence of 30% FCS and with medium alone served as positive and negative controls respectively. After overnight incubation of the transwell chamber at 37°C, 5% CO_2_, the upper side of the filters was carefully washed with cold PBS, and cells remaining on the upper face of the filters were removed with a cotton wool swab. Transwell filters were stained using Gill's haematoxylin, cut out with a scalpel, and mounted onto glass slides, putting the lower face on the top. Each experiment was performed in triplicate. Data were expressed as numbers of total migrated cells per insert after subtraction of the basal migration observed in the presence of DMEM alone (without presence of any growth factor). We investigated the influence of DA (1 µM) on chemotactic activity of 10 ng/ml VEGF [58].

### siRNA Transfection

The MSCs were trypsinized from their 3rd passage culture and were plated in 6-well plates at a density of 2×10^5^ cells/well in 2 ml antibiotic free normal growth medium (Murine Mesenchymal Stem Cell Expansion Medium; Millipore, USA) and allowed to attach overnight. The cells were then transfected with Akt siRNA (mice). Transfection was performed using siRNA transfection medium and siRNA transfection reagent (Santa Cruz Biotechnology) as per the manufacturer's instruction. Thereafter, following transfection, the cells were left to further grow for 48 hours and finally western blot was performed to confirm the silencing of the Akt using anti-Akt antibody (Santa Cruz Biotechnology) [59]. The concentration of siRNA was optimized to ensure that it did not affect cell viability.

To evaluate the role of the Akt in the migratory activity of MSCs, *in vitro* migration assay of siRNA transfected (Akt-silenced) MSCs were undertaken in transwell dishes (Corning Costar, Cambridge, MA) against chemotactic activity of 10 ng/ml VEGF.

### Western blot analysis to evaluate VEGFR-2 and Akt phosphorylation in MSCs

Anti-VEGFR-2, anti-p-VEGFR-2, anti-Akt and anti-p-Akt antibodies (Santa Cruz Biotechnology) were used as primary antibodies. Protein extracts from cells of various experimental groups were subjected to SDS-PAGE and then blotted onto PVDF membranes (Millipore, Bedford, MA). Protein loading was verified by stripping and reprobing the membrane with anti-VEGFR-2 and anti-Akt. Antibody-reactive bands were detected by enzyme-linked chemiluminescence (Pierce ECL, SuperSignal, Pierce Biotechnology) [59].

### Actin polymerization assay

Cells of various experimental groups were grown on glass coverslips and fixed in 37°C with 4% paraformaldehyde in PBS for 10 minutes. The cells were then incubated with 5 ml 0.15 M glycine solution for 5 minutes and permeabilized with a solution of 0.1% (v/v) Triton X-100 in PBS for 5 to 10 minutes. Finally, these cells were incubated with 1 µg/ml FITC-phalloidin (Sigma) staining solution for 40 minutes at room temperature [60].

### Statistical analysis

Data are means of at least 6 different experiments ± SEM. Student's t-test was used to analyze differences between groups. *p* value <0.05 was considered statistically significant [22].
